# Parenting Stress and Broader Phenotype in Parents of Children with Attention Deficit Hyperactivity Disorder, Dyslexia or Typical Development

**DOI:** 10.3390/ijerph16111878

**Published:** 2019-05-28

**Authors:** Paola Bonifacci, Laura Massi, Veronica Pignataro, Sara Zocco, Simona Chiodo

**Affiliations:** 1 Dipartimento di Psicologia, Università di Bologna, Viale Berti Pichat n.5, 40127 Bologna, Italy; veronica.pignataro@libero.it (V.P.); sara.zocco@gmail.com (S.Z.); 2 UO NPIA, Servi territoriali, Ausl Bologna, Via Sant’Isaia 90, 40123 Bologna, Italy; laura.massi@ausl.bologna.it (L.M.); simona.chiodo@ausl.bologna.it (S.C.)

**Keywords:** parenting stress, broader phenotype, endophenotypes, attention deficit hyperactivity disorder, dyslexia

## Abstract

In the present study parenting stress and the broader phenotype are investigated in two highly common developmental disorders, namely Attention Deficit Hyperactivity Disorder (ADHD) and specific reading impairment (dyslexia). Within a total sample of 130 parents, 27 were parents of children with ADHD (P-ADHD), 38 were parents of children with a diagnosis of dyslexia (P-DYS) and the other 65 participants were parents of children with typical development (P-TD). A battery of cognitive tasks was administered which included verbal and non-verbal Intellectual Quotient (IQ), reading speed (passage and nonwords), verbal fluency and the Attention Network Task (ANT). Reading history, symptoms of ADHD in adults and parenting stress were measured through questionnaires. Group differences evidenced that the P-DYS group had lower scores in the reading tasks, in the verbal fluency task and in the reading history questionnaire. Conversely, the P-ADHD group had more transversal cognitive weaknesses (IQ, reading tasks, verbal fluency) and the highest scores in parenting stress and ADHD symptoms, together with poor reading history. The groups did not differ in the ANT task. Parenting stress was predicted, on the whole sample, by lower socioeconomic status (SES) and number of family members and higher ADHD symptoms. Implications for research and clinical settings are discussed.

## 1. Introduction

In the past few decades a great amount of research led to an outstanding knowledge about the genetic, biological and neural basis of developmental trajectories in both typical and atypical populations. After Morton and Frith’s causal model [1], many studies investigated the etiology of developmental disorders on multiple levels of analysis (biological, cognitive and behavioral). Recent theoretical models, such as neuroconstructivism [2], reinforce the importance of considering how environmental variables, such as home literacy activities, parents’ education and parents’ cognitive skills, might interact with each of these levels. Robust evidence has shown that many neurodevelopmental disorders run in families and an increasing number of studies is addressing the issue of intergenerational transmission of risk and protective factors [3]. Parents’ might share with their offspring some specific cognitive markers and this is referred to as the so-called broader phenotype [4].

The concept of broader phenotype derives from the definition of endophenotypes (or intermediate phenotypes) [5], described as heritable neurophysiological, biochemical, endocrinological, neuroanatomical or neuropsychological constituents of disorders. One of the main characteristics of the endophenotypes is that they might be observable before the disease onset and, notably, in individuals with a heritable genetic risk for disease, such as unaffected family members (parents and siblings) [6]. The term broader phenotype refers specifically to the cognitive endophenotypes that can be observed in unaffected family members.

Thus, parents themselves might have similar cognitive weaknesses as their offspring, although at the behavioral and clinical level they might not meet criteria for diagnostic classification, or they did not undergo clinical evaluation in their life course. For example, parents of children with developmental dyslexia might themselves show weaknesses in phonological awareness skills, which is considered a clinical marker of reading impairment [4]. In the case of ADHD, if parents have low levels of response inhibition they might result to be impulsive in their interaction with the child and have difficulties in implementing daily routines. The concept of broader phenotype has received increasing attention in recent years because it directly poses an important question about the possible environment-child influence. That is, if parents themselves have some level of functional impairment in a set of specific cognitive functions, possibly the same that causes trouble in their offspring, how do they cope with their proper weaknesses? How do these weaknesses influence their parenting experience? In the present study, the broader phenotype of two highly common developmental disorders such as attention deficit hyperactivity disorder (ADHD) and specific reading impairment (dyslexia) is investigated both considering its usefulness in understanding the specificity of cognitive impairments within these families and evaluating how these cognitive weaknesses relate to parenting experience.

### 1.1. Dyslexia and ADHD: Two Common and Often Comorbid Neurodevelopmental Disorders

Dyslexia and ADHD are two of the most frequently occurring developmental patterns within the broader classification of neurodevelopmental disorders defined by DSM-5 [7]. Dyslexia is referred to as a specific learning disorder (SLD) that primarily affects the skills involved in accurate and fluent word reading and spelling. The latest definition given by DSM-5 [7] considers these disorders “specific” in that they are not primarily due to intellectual disability or global developmental delay, nor to neurological, motor or sensory disorders, or to a lack of opportunity of learning/inadequate instruction. Reading impairments may affect academic achievement or daily functioning if accommodations are not made. Consistent evidence has been collected that describes dyslexia as the behavioral outcome (poor reading fluency) of an underlying phonological deficit ([8]; for reviews), possibly associated with multiple risk factors [9]. In transparent orthographies, due to the high grapheme-phoneme consistency, reading impairment is better reflected by reading speed than by reading accuracy [10].

ADHD is a neurodevelopmental disorder whose main behavioral manifestations regard symptoms of inattention, impulsivity and hyperactivity, which occur in more than one social context (e.g., school, home) and significantly interfere with everyday life and social adaptability. These symptoms may resolve in adolescence or persist into adulthood, although the characteristics that mediate persistence or remission of ADHD during adulthood are still largely unexplored [11]. From a neurobiological perspective the putative endophenotypes considered to be core deficits of the disorder are referred to impairments in working memory, inhibition, cognitive control and time perception [12]. The Attention Network Task [13] is a two-choice reaction time (RT) task developed to independently and reliably test the efficiency of three attention networks, alerting, orienting and conflict resolution, which are theorized to be both anatomically and functionally segregated. The alerting network is thought to maintain the alerting state, the orienting network is hypothesized to allow the selection of sensory stimuli and the conflict network is designated to solve incongruent or competing stimuli. The strength of this task is specifically related to the data supporting high heritability for each attention network [14] and therefore has been suggested as a potential endophenotype of ADHD, with particular reference to the conflict network [15].

In sum, a neurobiological origin has been postulated both for ADHD and dyslexia, due to the interaction of genetic, epigenetic and environmental factors. Although different theoretical models have been proposed for each of these neurodevelopmental disorders, it is recognized that their aetiology is best explained within a multiple deficit model in which genetic factors interact with other risk factors connected with the pre- or perinatal period [9,16]. Furthermore, it has been proposed, based on their high comorbidity, that the two disorders share common cognitive deficits due to common genetic influences that increase susceptibility to both disorders [17].

### 1.2. The Broader Phenotypes of ADHD and Dyslexia

Various studies have ascertained that both ADHD and dyslexia run in families, with an increased probability for parents who have family risk for the disorder to have a child with clinical symptoms for that disorder: up to 66% for reading impairment [18,19] and up to 57% for ADHD [20].

Based on the intergenerational multiple deficit model [3] both parents convey risk and protective factors through interlaced genetic and environmental pathways and these factors ultimately impact on behavioral disorders or traits through complex interactions at the neural, cognitive and relational levels.

Signs of intergenerational transmission of executive functions (EF) have been examined in a number of studies, although with mixed results. Cuevas et al. [21] found moderate correlations (0.41) between maternal and preschool-aged child EF-task performance and this correlation pattern remained stable after controlling for maternal socioeconomic status (SES) and children’s verbal abilities. Goos et al. [22] found a significant correlation between children’s and parents’ response inhibition skills, that is the ability to inhibit a prepotent response, independently of ADHD symptom severity. Other studies, instead, have reported modest correlations between parents and children’s EFs [23]. In a recent study by Thissen et al. [24] the authors found significant parent-child correlations in EF and ADHD symptoms, parental ADHD was not associated with offspring EF or vice versa, thus there were no cross-correlations between EFs skills and ADHD symptoms in parents and children. Family studies have found further evidence of signs of broader phenotypes in the unaffected siblings of children with ADHD, with impairments in tasks assessing inhibition, verbal working memory and delay aversion [25,26]. As suggested by Deater-Deckard [27], parents’ own EFs skills might influence their caregiving behavior and, in turn, children’s behaviors and functioning might affect parents’ behavior. These reciprocal interactions should, therefore, be considered as candidate predictors of the quality of the parent-child relationship. However, following this undoubtedly valuable suggestion, family studies on ADHD under investigated other cognitive indexes of parents’ functional profile, such as for example reading related skills, which are known to be, at least in children, highly related to ADHD symptomatology.

The analysis of parent-child correlations in reading skills has been the focus of many family risk studies in the area of reading impairments. Black et al. [28] reported that mother’s, but not father’s, history of reading difficulties was correlated with child’s reading-related cognitive and behavioural scores. van Bergen et al. [29] showed that among children at family risk of dyslexia, parents of children who develop dyslexia underperformed in word reading fluency and letters and digits rapid automatized naming (RAN) compared to parents of children who did not develop dyslexia. Analogously, Torppa et al. [30] revealed that parents of children with reading impairments were poorer in reading and spelling accuracy, rapid word recognition, text reading fluency and vocabulary. Bonifacci et al. [31] found that parents of children with dyslexia differed from parents of children with typical development in all literacy measures (passage reading and accuracy, nonword reading, silent reading) and more frequently reported a history of poor reading. Recent evidence in the developmental pathway of children at family for dyslexia indicated that a family history of dyslexia is a predictor of literacy outcome from the preschool years. However, when children started formal schooling letter knowledge, phonological awareness, and RAN provided, beyond and above family risk, good sensitivity and specificity indexes of literacy achievement [16].

### 1.3. Parenting Stress in Parents of Children with ADHD or Dyslexia

Parenthood is an enriching experience, both from social and psychological perspectives, but it also constitutes a crucial transition due to the specific demands that an individual needs to manage in a sufficiently adequate manner. Parenting stress has been defined as *“the stress reaction to the demands of being a parent*” ([32], pp.314) and its determinants can be ascribed to parent characteristics, life stress and socio-demographic factors, and child characteristics [33]. A perceived discrepancy between parents’ perception of his or her resources and the demands they are exposed to having a child might increase levels of parenting stress. There is evidence that parenting stress influences parenting practices and, as a consequence, child development ([32], for a review).

Being a parent of a child with neurodevelopmental disorders might challenge the perception to fulfill adequately these demands and therefore might increase the psychological cost of parenthood. This might be related to at least two main factors: (1) the child characteristics, which, due to the cognitive and behavioural impairments, might increase the demands and therefore the cost of parenthood; (2) parents’ cognitive and psychological resources. Based on the literature previously discussed, parents of children with neurodevelopmental disorders might themselves have weaknesses at the cognitive and behavioural levels, often in the same areas of those manifested by their offspring. This, in turn, might amplify their perception of being inadequate in their parental role, particularly if their own weaknesses had never been appropriately recognized and treated in their life course. In the literature, higher levels of parenting stress have been documented in parents of children with a wide range of developmental disorders, included ADHD and dyslexia.

As far as ADHD was concerned, a meta-analysis reviewed research studies on parenting stress in families of children with ADHD (age 12 or younger) and showed that parents experienced much higher levels of parenting stress compared to parents of typically developing children [34]. Similar results have been found in parents of adolescents with ADHD [35].

With reference to SLD, there is still a paucity of research on parenting stress. Some studies were conducted on parents of children with mild or moderate intellectual dysfunctions and higher levels of parenting stress were found [36], together with higher levels of child monitoring [37], and worries about their children’s future [38]) in mothers of children with learning disabilities. Considering more precisely parents of children with *dyslexia*, [39] observed that about 74% of parents reported that the child’s disorder has had negative effects on family life and that mothers reported high anxiety and/or depression levels (see [40], for similar results on anxiety symptoms). Two recent studies specifically addressed the issue of parenting stress in parents of children with SLD. In the first study, [31] found that parents of children with dyslexia reported higher scores in the perception-child dysfunctional interaction scale and in the difficult child scale. The second study [41], involving children with SLD in comorbidity (at least two impairments, either in reading, writing, or calculation abilities) evidenced higher levels of parenting stress in all scales of the Parenting Stress Index [33]. In both of the latter studies, there were no differences between mothers and fathers.

To conclude, this introduction evidenced two main points. On the one hand, there is increasing evidence about the broader phenotype of developmental disorders, which suggests that parents of children with neurodevelopmental disorders might share cognitive and behavioural weaknesses with their offspring. On the other hand, a vast literature evidenced that parents of children with neurodevelopmental disorders such as ADHD and dyslexia, have higher levels of parenting stress compared to parents of children with typical development. However, scarce evidence has been collected on the interplay between parents’ cognitive profiles and parenting stress in populations of children with ADHD and dyslexia.

### 1.4. The Present Study

The aims of the present study were threefold:

(1) To investigate group differences in cognitive and behavioral indexes of reading and attention impairments comparing three groups of parents: parents of children with ADHD (P-ADHD), parents of children with developmental dyslexia (P-DYS) and parents of children with typical development (P-TD). This first objective was intended to verify if the three groups of parents did indeed show endophenotypic patterns specific for their offspring developmental profile. Based on the literature review, P-DYS are expected to fail in reading related measures, whereas P-ADHD are expected to differ compared to the other groups in ANT task and in behavioural scales assessing ADHD symptoms in adulthood. Differences between mothers and fathers will be considered.
(2) To investigate group differences in parenting stress. Based on previous studies higher levels of parenting stress have been reported for parents of children with dyslexia and parents of children with ADHD compared to parents of typically developing children. We therefore expect replying these findings and evaluating, for the first time, differences in parenting stress between parents of children with dyslexia or ADHD.
(3) To analyse the relationships between cognitive indexes and parenting stress in the whole sample. Specifically, through a step-wise regression model the study aimed at evaluating which factors better predicted parental stress, an important index assessing parent-child interactions and known to correlate with parental styles. Cognitive measures are expected to significantly predict parenting stress, based on the assumption that parenting stress depends on parents’ ability to cope cognitively with offspring’s requests.

## 2. Methods

### 2.1. Participants

The study was conducted on a total sample of 130 parents (68 mothers and 62 fathers), with a mean age of 43.8 years (5.08 SD). Within the total sample, 38 were parents of children with a diagnosis of dyslexia (P-DYS) (mean age: 43.65 ± 5.08 years); 27 were parents of children with ADHD (P-ADHD) (mean age: 43.33 ± 5.7 years). The other 65 participants (mean age: 44.12 ± 4.98) were parents of children with typical development (P-TD). Parents of children with ADHD were recruited at the Maggiore Hospital, Department of Developmental Psychiatry and Psychology, in the city of Bologna. Children underwent a full clinical evaluation by a multidisciplinary team and, according to ICD-10 criteria, they received a diagnosis of ADHD (F 90.0), in the absence of comorbidity with dyslexia. The P-DYS group was recruited at LADA lab (Laboratory for the Assessment of Learning Disorders), Department of Psychology, University of Bologna. To be included in the study, they had to be parents of a child who received a diagnosis of specific reading disorder (ICD-10 code: F 81) in the last 2 years. None of the children with dyslexia had comorbidity with ADHD. The P-TD group was recruited through leisure centers and through word of mouth. None of the children of the control group had been previously referred for showing risk factors for dyslexia or ADHD. All parents fulfilled inclusion criteria as follows: being biological parents; free of psychiatric disorder; Italian monolinguals, family not in care of social services; intellectual functioning within the normal range (Total IQ > 70). Independently of their civil state (married or not), we asked both parents to take part in the study. Depending on their willingness to volunteer, for some children only one parent was included in the study. In the final sample there were 60 couples participating and 10 single parents (2 in the P-DYS group, 7 in the P-TD group and 1 in the ADHD group) (**χ**^2^(2) = 1.78, *p* = 0.4). Participants volunteered for the study and signed the informed consent and data treatment documents before starting. This study was carried out in accordance with the recommendations of American Psychological Association’s Ethical Principles (1982), and the research ethics committee of the AUSL of Bologna approved the project (Prot. N.559/CE, Cod. CE: 12020).

### 2.2. Measures

#### 2.2.1. Background Information

Both parents were asked to fill out a short questionnaire that included socio-demographic information, such as SES (calculated based on the Four Factor Index of Social Status, Hollingshead, 1975), civil state, and evaluation of previous scholastic achievement. Parents were also asked to evaluate their children scholastic achievement (reading, math, writing, science, grammar, history) and to indicate the number of family members.

#### 2.2.2. Cognitive Measures

(1) Kaufman Brief Intelligence Test-2. ([42]; Italian version adapted and standardized by [43]). This test assesses intellectual functioning and comprises Vocabulary (verbal knowledge and riddles) and Matrices subtests. It gives standardized measures of Verbal (VIQ), Performance (PIQ) and Composite Full Scale IQ.
(2) Passage reading, taken from the “Reading tasks for the secondary schools” [44]. Participants were asked to read aloud a passage (729 words) and reading speed (syllables per second) and accuracy (number of errors) were recorded.
(3) Non-word reading task, taken from the “Battery for the assessment of developmental dyslexia and dysorthographia” [45]. Participants are required to read aloud a list of 48 nonwords and reading speed (syllables per second) was recorded.
(4) *Semantic fluency:* The participants were asked to produce as many words within the same semantic category (professions) in a test interval of 1 min.
(5) Attentional Network Task (ANT) [13]. This task is a combination of a cue reaction time task, and a flanker task exploring attentional abilities divided into three components: executive control (conflict resolution), alerting and orienting. It requires participants to indicate whether a central arrow is oriented to the right or left. The arrow is presented between flanker arrows pointing either in the same direction (🡢🡢🡢🡢🡢; congruent condition) or in different directions (🡢🡢🡠🡢🡢; incongruent condition) from the target. Responses are expected to be slower for incongruent than for congruent conditions, showing that more cognitive effort is needed to resolve the conflict. The alerting component is explored by showing that faster responses occur when a cue is presented before the target stimulus compared to when it is not. Finally, orienting is studied by showing that responses are faster when a cue indicates the position of a target stimulus compared when it does not. The presentation of the stimuli was as follows: (a) a fixation point (**+**) appeared on the center of the screen for 400 ms; (b) a cue (*) was presented for 100 ms; (c) a fixation period was provided for 400 ms after the cue; (d) the target arrow and the flankers were presented simultaneously until the participant’s response or up to 1700 ms, (f) the target and flankers disappeared after response and the next trial began. Participants were instructed to focus on the fixation point and to respond by pressing a key on the computer keyboard, as quickly and accurately as possible, with their left hand when the arrow pointed to the left and with the right hand when it pointed to the right. A training phase consisting of 24 trials was administered.

### 2.3. Questionnaires

(1) Adult Reading History Questionnaire-Revised (ARHQ-R) [46]: The ARHQ-R is aimed at evaluating the presence of a significant history of reading difficulties. Parents are required to respond to twenty-three questions on a five-point Likert scale (from 0 to 4), with higher values corresponding to more problems with reading skills, less print exposure, or poorer attitude towards reading. The participant’s score was calculated by dividing the total score by the maximum possible score (92). A score above 0.30 is indicative of a positive history of reading disorders.
(2) Adult ADHD Self-Report Scale (ASRS-v1.1) Symptom Checklist. The Symptom Checklist is an instrument based on DSM-IV-TR criteria for ADHD, developed by World Health Organization (WHO). It includes 18 questions (e.g., How often do you have difficulty getting things in order when you have to do a task that requires organization?), based on a five points Likert-scale.
(3) Parenting Stress Index (PSI) [33]. The PSI is addressed to the evaluation of parenting and family characteristics with the aim of identifying indexes of parental behavior problems and child adjustment difficulties within the family system. In this work we used the Italian PSI Short Form (PSI/SF), it includes 36 items (e.g., My son often wakes up in a bad mood) and yields a Total Stress score from three scales: Parental Distress, Parent-Child Dysfunctional Interaction, and Difficult Child. It also provides a Defensive Response scale. Higher scores are representative of higher levels of parenting stress.

### 2.4. Data Analysis

In order to test group differences in background information, we performed a set of ANOVAs applying Bonferroni post-hoc tests. For categorical variables, we tested differences in groups’ distribution through Chi-Square (χ) analysis. Then a set of ANOVAs and MANOVAs were run with Group (P-DYS, P-ADHD, P-TD) and Role (parents’ gender: Mothers, Fathers) as independent factors and scores at cognitive tasks and questionnaires as dependent variables. Finally, in order to investigate which factors predicted Parenting Stress a regression model was run. In the first step SES, parents’ age and number of family members were included, in the second step cognitive variables were included (Composite IQ, Verbal fluency, non word reading speed, attentional networks - conflict), and the third step included behavioral measures of reading history and ADHD symptoms in parents.

## 3. Results

### 3.1. Background Information

A summary of mean values related to background information is reported in Table 1.

The three groups did not differ for children’s age (F(2,130) = 2.87; *p* = 0.06, η^2^ = 0.04), parents’ age (F(2,130) = 0.26; *p* = 0.7, η^2^ = 0.0) and civil state (χ^2^(4) = 4.6, *p* = 0.33); more than 85% of parents in each group were married or living together. There was instead a difference in mean SES (F(2,122) = 8.08; *p* < 0.001, η^2^ = 0.12), with the P-TD group showing higher values compared to the other two groups (*p* < 0.01), which did not differ from each other. The P-TD group also reported to have reached brilliant scholastic results in a higher percentage of cases (58.5%) than the P-DYS (41.1%) and the P-ADHD group (χ^2^(4) = 17.48, *p* < 0.01). Families in the ADHD group reported to have a lower number of children compared to families in the TD-group, who, in turn, had less children than families in the P-DYS group (F(2,123) = 13.02; *p* < 0.001, η^2^ = 0.17).

Considering parents evaluation of their offspring scholastic achievement, the MANOVA showed a main effect of group (F(12,232) = 5,74; *p* < 0.001, η^2^ = 0.23). It emerged that children in the P-DYS group were considered to have lower performance compared to the TD group in all disciplines considered. The ADHD group differed from the control group only in Writing skills (*p* < 0.01) and children with ADHD were reported having better scores in reading (*p* < 0.001) and grammar (*p* < 0.05) compared to the group of children with dyslexia.

### 3.2. Cognitive Measures

In Table 2 a summary of mean values for cognitive tasks is reported. The MANOVA with Group (P-DYS, P-ADHD, P-TD) and Role (parents’ gender: Mothers, Fathers) as independent factors and VIQ and NVIQ as dependent variables, showed a main effect of group (F(4,248) = 4.6; *p* = 0.001, η^2^ = 0.07). There was no effect for Role (F(2,123) = 2.11; *p* = 0.12, η^2^ = 0.03), nor for the interaction Group*Role (F(4,248)=2.03; *p* = 0.09, η^2^ = 0.03). Univariate analyses and Bonferroni post-hoc comparisons showed that the P-ADHD group had significantly lower scores compared to the P-TD group in both Verbal (*p* = 0.001) and Non Verbal IQ (*p* = 0.001).

Considering Verbal Fluency, the ANOVA showed a main effect of Group (F(2,129) = 12.04; *p* < 0.001, η^2^ = 0.16) and a marginal effect of Role (F(1,129) = 3.9; *p* = 0.05, η^2^ = 0.03), but the interaction Group*Role was not significant (F(2,129) = 0.4; *p* = 0.67, η^2^ = 0.006). Parents of children with TD outperformed compared to both P-ADHD (*p* < 0.001) and P-DYS (*p* < 0.01). Mothers tended to produce more words than fathers (*p* = 0.5).

**Figure 1:**
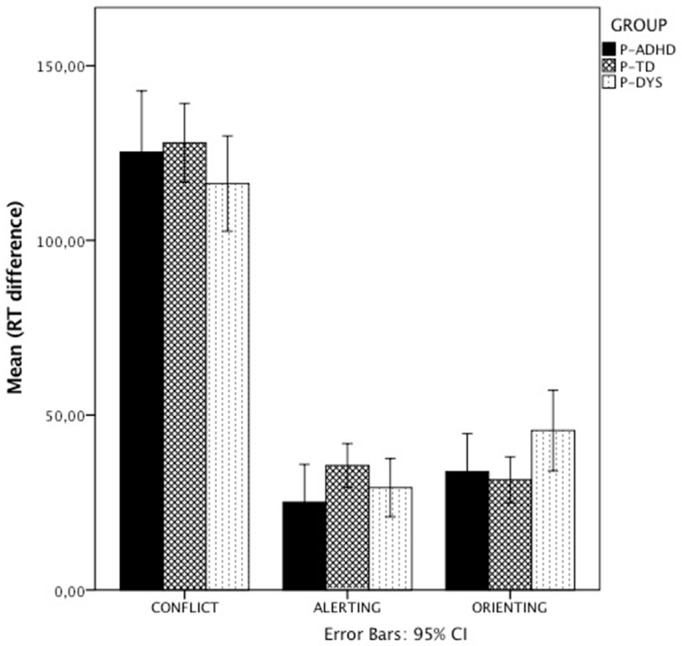
Magnitude (ms) of the three effects of the ANT task broken by group of participants. Error bars represent 95% CI.

As far as reading measures were concerned, the MANOVA on passage and nonword reading speed showed a main effect of Group (F(4,466) = 7.06; *p* < 0.001, η^2^ = 0.10). Both univariate analyses were significant (Passage: F(2,128) = 17.41; *p* < 0.001, η^2^ = 0.2; Nonwords: (F(2,128) = 8.93; *p* < 0.001, η^2^ = 0.13) and Parents in the DYS group were slower compared to the P-TD group in both tasks (*p* < 0.001), whereas P-ADHD differed from the P-TD group only in passage reading speed (*p* < 0.05). Finally, to analyze results from the ANT task we performed two MANOVAs, one on accuracy and the other on RT parameters, see Figure 1.

In both analyses the main factor Group did not result to reach statistical significance (Accuracy: (F(6,220) = 0.54; *p* = 0.78, η^2^ = 0.01; RT: (F(6,220) = 1.82; *p* = 0.096, η^2^ = 0.047), nor it was for Role (Accuracy: (F(3,109) = 1.11; *p* = 0.35, η^2^ = 0.03; RT: (F(3,109) = 0.3; *p* = 0.8, η^2^ = 0.01) or for the interaction Group*Role (Accuracy: (F(6,220) = 0.65; *p* = 0.68, η^2^ = 0.017; RT: (F(6,220) = 0.3; *p* = 0.8, η^2^ = 0.01).

### 3.3. Questionnaires

In Table 2 a summary of mean values for questionnaires’ scores is reported. Taking into account the Adult Reading History Questionnaire the ANOVA revealed a main effect of group (F(2,123) = 11.2; *p* < 0.001, η^2^ = 0.16), with Bonferroni post-hocs showing that both P-DYS (*p* < 0.001) and P-ADHD (*p* < 0.01) reported more problems in reading history and attitude compared to the P-TD group. There was no effect of Role (F(1,123) = 0.0; *p* = 0.9, η^2^ = 0.0), nor any significant interaction Group*Role (F(2,123) = 1.57; *p* = 0.21, η^2^ = 0.02).

There was a main effect of Group (F(2,123) = 5.19; *p* < 0.001, η^2^ = 0.08) also in the ASRS Questionnaire addressing ADHD symptoms in adulthood. Bonferroni post-hoc comparisons revealed the P-DYS group did not differ from either the P-TD (*p* = 0.27) group or the P-ADHD group (*p* = 0.33). Instead, the P-ADHD group showed higher scores (*p* < 0.01) compared to the P-TD group. There was no effect of Role (F(1,123) = 0.52; *p* = 0.47, η^2^ = 0.004), nor of Group*Role interaction (F(2,123) = 1.2; *p* = 0.3, η^2^ = 0.02).

Finally, considering the Parenting Stress Index, an ANOVA was run on the Total score and a main effect of Group arose (F(1,123) = 20.23; *p* < 0.001, η^2^ = 0.25), but there was no effect of Role (F(1,123) = 0.06; *p* = 0.8, η^2^ = 0.001), or of the interaction of Group*Role (F(1,123) = 0.11; *p* = 0.89, η^2^ = 0.002). Bonferroni post-hoc comparisons showed that the P-ADHD group had higher scores both compared to the P-TD (*p* < 0.001) and P-DYS (*p* < 0.001) groups, which did not differ each other (*p* = 0.3). The same pattern was observed when considering the single subscales (Defensive Scale, Parental Distress, Difficult Child, Difficult Child Interaction), where P-ADHD participants always obtained higher scores compared to the other two groups.

### 3.4. Regression Model

In order to investigate which factors predicted Parenting Stress a hierarchical regression model was run. Results are presented in Table 3. The results of the analysis showed that, at first step, SES and number of family members, but not parents’ age, were significant predictors, explaining 13% of variance. At the second step, parents’ cognitive skills did not add a significant contribution to the model (variance explained: 18%). At the final step, when parents’ reading history and symptoms of adults’ ADHD were added the model significantly increased in the portion of variance explained (27%) and in the final model it emerged that lower SES (*β* = −0.24, *p* < 0.05), minor number of family members (*β* = −0.23, *p* < 0.05) and higher ASRS score (symptoms of ADHD in parents) (*β* = 0.299, *p* < 0.01) were significant predictors of higher parenting stress.

## 4. Discussion

The present study investigated the cognitive and behavioral profiles of parents of children with neurodevelopmental disorders, specifically ADHD and dyslexia, and with typical development. The aims were threefold. First, we wanted to evaluate the broader phenotype of ADHD and dyslexia by assessing putative endophenotypes of these disorders within family members (parents). Secondly, we wanted to test differences in levels of parenting stress amongst the three groups. Finally, the study aimed to test which cognitive and behavioural indexes better predicted parenting stress. Considering the first aim, group differences on demographic, cognitive and behavioural variables depicted a complex picture of overlapping and distinct features amongst the three groups considered. Both P-ADHD and P-DYS parents had lower SES compared to parents of typically developing children, but the three groups did not differ in civil state, parents’ and children’s age. The difference in SES between P-ADHD and P-TD groups is quite consistent with data reported in the literature (e.g., [35]), and in our sample, it was consistent for both mothers and fathers. Data on SES in the P-DYS group are in line with van Bergen and colleagues [29,47], who found differences between at-risk and control families in the educational level.

Moving to the cognitive profile, it emerged that the P-ADHD differed from the P-TD group in both verbal and non verbal IQ whereas the P-DYS did not differ from the other two groups. Furthermore, both the P-DYS and P-ADHD group significantly underperformed compared to the P-TD group in verbal fluency and passage reading speed, whereas in nonword reading speed it was the P-DYS group that underperformed compared to the P-TD group. There were no differences between the three groups in the ANT task, either considering accuracy or speed parameters.

When considering the questionnaires assessing behavioural symptoms of ADHD and troubles in reading history, it emerged that both P-DYS and P-ADHD groups reported a significant poor reading history compared to P-TD group. Instead, in the ASRS questionnaire, only the P-ADHD group showed consistent signs of ADHD symptoms.

Finally, when analyzing parenting stress, the P-ADHD group resulted to report the highest level in all dimensions considered: parental distress, defensive response, difficult child, difficult child-interaction. In contrast, the P-DYS group did not differ from the P-TD group, although mean values tended to be slightly higher in DC and DCI scales

Taken as a whole, these results evidence some aspects of specificity with regards to the two distinct profiles analyzed, i.e., ADHD and dyslexia. In particular, the group of parents of children with dyslexia show a profile quite similar to what is reported in the literature for children with dyslexia: they show a fully adequate intellectual functioning, similar to that of parents of children with typical development. Nevertheless, they present specific weaknesses in reading speed, notably they underperform compared to the other two groups in reading nonwords, which is a task assessing the phonological basis of decoding skills. These weaknesses are also associated with poorer reading history, remarkably with mean scores in the clinical range. They do not differ from the other groups in behavioral symptoms of ADHD, suggesting that for these parents ADHD symptomatology is not crucially associated to their profile and its occurrence might be explained as an epiphenomenon of their cognitive profile [48]. This is in line with their adequate performances in the ANT task assessing executive functions and attentional networks. Interestingly, in this study, and differently from other evidence in the literature, they did not result to have a higher level of parenting stress. These relatively modest discrepancies in parenting stress might be due to the differential composition of the control group, because, actually, the mean values of the P-DYS group are in the same range of those observed in previous studies [31,41]. As outlined by [32] parenting stress is a dimensional variable that occurs on a continuum, on which probably parents of children with dyslexia are not positioned at the upper extreme, but rather in a medium to high range, depending on a number of different variables which would deserve further attention in future investigations.

The profile of parents of children with ADHD is instead much less clear-cut. They seem to have more transversal cognitive weaknesses involving intellectual functioning, word reading speed and verbal fluency. However, they do not show specific impairments in attention networks, and do not differ from P-TD group on a phonological based measure such as non-word reading. At the behavioural levels, they report both reading history troubles and adult ADHD symptoms, the latter being significantly higher compared to those of parents of typically developing children. They also reported the highest level of parenting stress compared to the other groups. The higher incidence of ADHD symptomatology in parents of children with ADHD confirms previous data [49] and reinforces the idea that ADHD runs in families, although, according to [24], non-EF factors might play a crucial role in the intergenerational model of ADHD aetiology, challenging the concept of EF as core endophenotypes of ADHD.

The present study also considered the parental role (fathers vs mothers) and it was found that, except for a tendency for mothers to have better verbal fluency skills compared to parents, no other significant differences emerged in demographic, cognitive and behavioral indexes considered, and none of the group*role interactions resulted to be significant. This trend might be considered as indirect support of the non-random mating hypothesis [3,48], with both parents within each group showing very similar characteristics.

Finally, considering the predictors of parenting stress at the cognitive and behavioural level it emerged that behavioral (ADHD symptoms in parents) and demographic (SES, number of family members) significantly predicted parenting stress, within a model that explained around 27% of variance. Thus, parents with higher levels of ADHD symptoms, lower SES and smaller family sizes are more susceptible to higher levels of parenting stress. Instead, markers of parents’ cognitive profile did not seem to be crucially associated to how parents cope with the demands of their role. This aspect would deserve further investigation in future studies, since, to our knowledge, this is an under-investigated area.

This study has a number of limitations that might limit the generalizability of results. First of all, the sample size, which, particularly for the ADHD group, is relatively modest. Furthermore, the selection of markers of the broader phenotypes for the two clinical groups considered might have included other measures such as paradigms of delay aversion and time perception, which have been advocated for being important endophenotypic measures of ADHD. Referred to the broader phenotype of dyslexia further studies might include more specific measures of phonological awareness that are considered as putative markers of the broader phenotype of dyslexia [4,31]. Finally, it would be of interest to include in future studies samples of children with comorbid ADHD and dyslexia. Notwithstanding, this is, to our knowledge, the first study linking measures of cognitive functioning to parenting stress in a cross-group comparison involving parents of children with two different clinical profile and parents of children with typical development and the results reported shed important insight for future research in this new research area.

## 5. Conclusions

Considering the main aims of the study the paper offered a rich pattern of findings. As far as parent’s group differences in cognitive profile were concerned the study highlighted that parents of children with ADHD had transversal cognitive weaknesses (IQ, reading tasks, verbal fluency), whereas the P-DYS group showed more specific falls in the reading related tasks. Turning to the second aim, it emerged that the P-ADHD had the highest levels of parenting stress and ADHD symptoms. Finally, the main predictors of parenting stress resulted to be a lower SES together with a minor number of family members and higher levels of ADHD symptoms in parents.

Lastly, some clinical implications might be proposed. First of all, the fact that parents of children with dyslexia or ADHD might themselves show some cognitive weaknesses that are similar to those of their offspring needs to be taken seriously into account when clinicians suggest intervention programs and, especially, when they recommend parental best practices for dealing with children impairments. Actually, many parents might encounter significant difficulties in fulfilling these requirements because they have problems similar to those of their children and might not be endowed with the necessary cognitive and psychological resources to accomplish clinician requirements. On the other hand, to see the glass half full, clinicians might valorize parents’ experience trying to understand how they have afforded difficulties in their life course, what themselves would have or have found helpful, and treasure their knowledge of their children habits and demands. Within a systemic perspective, it would be important to accurately consider strengths and weaknesses within the family system, avoiding transmitting feeling of guil in parents who actually already perceive themselves as non-sufficiently adequate in managing parenthood demands.

## Figures and Tables

**Table 1:** Means and standard deviation for demographic variables and parents evaluation of children scholastic achievement. for the three groups (P-ADHD, P-TD, P-DYS).

Measures	P-ADHD		P-TD		P-DYS		*p*-Values	Bonferroni Post-Hoc Comparisons
	Mean	SD	Mean	SD	Mean	SD		
Children age	9.3	1.5	9.8	1.8	10.3	1.7	0.06	NS
Parents age	43.3	5.7	44.1	5	43.7	4.9	0.7	NS
SES	35.24	11.04	43.82	9.32	36.68	12.42	<0.001	TD > DYS = ADHD
Reading *	2.05	0.38	2.26	0.44	1.45	0.6	<0.001	TD = ADHD > DISL
Writing *	1.81	0.68	2.25	0.43	1.61	0.64	<0.001	TD > DYS = ADHD
Grammar *	1.95	0.5	2.25	0.53	1.5	0.6	<0.001	TD = ADHD > DYS
History *	2.05	0.5	2.37	0.55	1.89	0.77	<0.001	TD > DYS
Mathematics *	1.9	0.77	2.28	0.55	1.58	0.76	<0.001	TD > DYS
Science *	2.1	0.54	2.29	0.52	1.84	0.69	<0.001	TD > DYS

* Parents’ evaluation of children’s skills. Calculated based on Hollinshead formula.

**Table 2:** Means and standard deviations for cognitive tasks and questionnaires’ scores for the three groups (P-ADHD, P-TD, P-DYS).

Measure	P-ADHD		P-TD		P-DYS		*p*-Values	Bonferroni Post-Hoc Comparisons
	Mean	SD	Mean	SD	Mean	SD		
Verbal IQ	100.10	7.50	108.40	9.60	105.20	10.30	<0.001	TD > ADHD DYS = ADHD; DYS = TD
Non Verbal IQ	96.80	17.00	108.90	13.80	104.70	11.80	<0.001	TD > ADHD; DYS = ADHD; DYS = TD
Semantic Fluency	14.70	3.40	19.20	4.60	16.20	4.50	<0.001	TD > ADHD = DYS
Passage reading speed (syll/sec)	5.38	1.21	6.10	1.04	4.91	0.98	<0.001	TD > ADHD = DYS
Non word reading speed (syll/sec)	2.65	0.76	3.03	0.71	2.41	0.69	<0.001	TD > DYS = ADHD
ARHQ Total	0.35	0.11	0.26	0.09	0.37	0.17	<0.001	TD > ADHD = DYS
ASRS Total	6.76	4.34	3.91	3.18	5.13	4.06	<0.01	ADHD > DYS = C
* PSI Defensive Scale	1.08	1.31	−0.08	0.95	−0.03	1.15	<0.001	ADHD > DYS = C
PSI Parent distress	0.96	1.34	−0.25	0.86	−0.15	1.11	<0.001	ADHD > DYS = C
PSI Difficult Child Interaction	1.49	1.77	0.12	0.88	0.62	1.30	<0.001	ADHD > DYS = C
PSI Difficult Child	1.74	1.58	0.21	0.96	0.57	1.06	<0.001	ADHD > DYS = C
PSI Total Score	1.76	1.42	0.02	0.83	0.39	1.22	<0.001	ADHD > DYS = C

* For Parenting Stress Index z scores are reported.

**Table 3:** Hierarchical regression; dependent variable: Parenting Stress Total.

Step	Measure	B	SE B	β
1 (R^2^ = 0.137)	SES	−0.6	0.176	−0.31 **
	Number family members	−4.679	1.677	−0.25 **
	Parents’ age	0.008	0.395	0.002
2 (ΔR^2^ = 0.047. *p* = 0.21)	SES	−0.481	0.193	−0.251 *
	Number family members	−4.818	1.777	−0.264 **
	Parents’ age	−0.027	0.398	−0.006
	Non-word reading speed	−3.493	2.958	−0.116
	Verbal fluency	−0.67	0.432	−0.157
	ANT-Conflict (RTs)	0.024	0.046	0.046
	Composite IQ	0.001	0.189	0.001
3 (ΔR^2^ = 0.084. *p* < 0.01) (R^2^ = 0.268)	SES	−0.453	0.197	−0.236 *
	Number family members	−4.23	1.709	−0.231 *
	Parents’ age	−0.072	0.382	−0.017
	Non-word reading speed	−3.968	2.949	−0.132
	Verbal fluency	−0.581	0.415	−0.137
	ANT-Conflict (RTs)	0.019	0.045	0.037
	Composite IQ	0.076	0.182	0.042
	ARHD (reading history)	0.083	17.701	0
	ASRS (ADHD symptoms)	1.771	0.534	0.299 **

* *p* < 0.05; ** *p* < 0.01.
